# Reforming primary health care: is New Zealand's primary health care strategy achieving its early goals?

**DOI:** 10.1186/1743-8462-5-24

**Published:** 2008-11-06

**Authors:** Jacqueline Cumming, Nicholas Mays, Barry Gribben

**Affiliations:** 1 Health Services Research Centre, School of Government, Victoria University of Wellington, Wellington, New Zealand; 2 Health Services Research Unit, Department of Public Health and Policy, London School of Hygiene and Tropical Medicine, London, UK; 3 CBG Health Ltd, Auckland, New Zealand

## Abstract

**Background:**

In 2001, the New Zealand government introduced its Primary Health Care Strategy (PHCS), aimed at strengthening the role of primary health care, in order to improve health and to reduce inequalities in health. As part of the Strategy, new funding was provided to reduce the fees that patients pay when they use primary health care services in New Zealand, to improve access to services and to increase service use. In this article, we estimate the impact of the new funding on general practitioner and practice nurse visit fees paid by patients and on consultation rates. The analyses involved before-and-after monitoring of fees and consultation rates in a random sample of 99 general practices and covered the period from June 2001 (pre-Strategy) to mid-2005.

**Results:**

Fees fell particularly in Access (higher need, higher per capita funded) practices over time for doctor and nurse visits. Fees increased over time for many in Interim (lower need, lower per capita funded) practices, but they fell for patients aged 65 years and over as new funding was provided for this age group. There were increases in consultation rates across almost all age, funding model (Access or Interim), socio-demographic and ethnic groups. Increases were particularly high in Access practices.

**Conclusion:**

The Strategy has resulted in lower fees for primary health care for many New Zealanders, and consultation rates have also increased over the past few years. However, fees have not fallen by as much as expected in government policy given the amount of extra public money spent since there are limited requirements for practices to reduce patients' fees in line with increases in public funding for primary care.

## Introduction

In 2001, the New Zealand government introduced its Primary Health Care Strategy (PHCS), aimed at strengthening the role of primary health care, in order to improve health and to reduce inequalities in health [1]. Implementation of the Strategy has involved three main changes in policy. First, the government has provided significant additional funding in order to reduce the fees that patients pay when they use primary health care services, and to encourage the development of new services. Second, the Government has encouraged the development of new organisations, Primary Health Organisations (PHOs), to plan and provide some primary health care services at a local level [2]. PHOs are local, non-governmental, not-for-profit organisations, contracted by their local District Health Board (DHB) [3] to manage primary health services for their enrolled populations. Third, public funding of primary health care has changed from fee-for-service subsidies at the practitioner level to capitation funding of PHOs with remaining patient fees still paid out-of-pocket to practices.

As a result of the Strategy, New Zealand now has 80 PHOs overseeing the planning and delivery of primary health care services; government spending to support primary health care services is now higher than before; and all New Zealanders enrolled with PHOs are now eligible for some public funding for primary health care services. Overall, the government aims to allocate around $2.2 billion over seven years from 2002/03 to support the introduction of the Strategy. (On 26 September 2008, the New Zealand dollar was worth: $US 0.70; $Australian 0.83; £Stirling 0.38; Euro 0.48.)

In this article, we explore the impact of the changes brought about by the PHCS, focusing in particular on the changes in the fees that New Zealanders pay when they access primary health care services, changes in consultation rates for primary health care services, and the impact of these changes on different population groups, with a view to exploring the implications of the Strategy in terms of improving health and reducing inequalities in health in the future.

## Background

Prior to the introduction of the PHCS, the New Zealand government provided partial, targeted, fee-for-service subsidies for visits to general practitioners (GPs), with around half the New Zealand population eligible for such support. Access to subsidised care was provided for all children under six years of age, with subsidy rates ($32.50 per visit in 2002) expected to cover the full cost of services provided to children. For young people aged 6–17 and for adults, partially subsidised care was available to those with subsidy cards, either a community services card (CSC), available to those families on lower incomes, or a high user health card (HUHC), available for people who had an on-going health condition and who had visited the GP 12 or more times in the previous 12 months. For young people, subsidies of $15 and $20 were available respectively for those with and without subsidy cards; for adults, subsidies of $15 per visit were available for those with cards. In most cases, people with subsidy cards also paid a fee to their primary health care provider. Adults without a subsidy card paid the full cost of primary health care themselves, which could vary, at the GP's discretion, from nothing to around $60 for a standard GP consultation in 2002 before the Strategy began to be implemented.

This approach based on fee-for-service government subsidies coupled with patients paying unregulated fees has been criticised for many years, particularly on the grounds that it contributed to poor access to first contact care for some groups in the population, arising from financial, as well as cultural and other barriers [4-9]. Surveys undertaken by the Commonwealth Fund in 1998 and 2001, for example, found that 20% of New Zealanders reported financial barriers to getting primary medical care, with statistically significantly higher rates for those on below-average incomes [10,11]. The New Zealand Health Survey showed that 12% of New Zealanders reported that they needed to see a GP, but did not do so in 2002/03, 48% of these due to the cost of a GP consultation. The rate of unmet need was higher for particular groups in the community, such as Maori (21%) and Pacific groups (17%) and those in lower socio-economic areas (quintiles 4 and 5 14% and 15%, respectively) [12].

The Primary Health Care Strategy aims to achieve a new vision of primary care over five to ten years in which, "people will be part of local primary health care services that improve their health, keep them well, are easy to get to and co-ordinate their ongoing care" and which, "will focus on better health for a population and actively work to reduce health inequalities between different groups" [1].

Six key policy directions support the vision: work with local communities and enrolled populations; identify and remove health inequalities; offer access to comprehensive services to improve, maintain and restore people's health; co-ordinate care across service areas; develop the primary health care workforce; and continuously improve quality using good information [1].

Key priorities for early action were to reduce barriers, particularly financial barriers, to the use of services for the population groups with the greatest health need; support the development of PHOs; encourage multi-disciplinary approaches to services and decision-making; support the development of services by Maori and Pacific providers; and educate the public about enrolment and PHOs [1].

In terms of the funding of primary health care, the PHCS signals a move away from a targeted approach where the government only provides funding to support access to primary health care for some New Zealanders to a more universal approach where all New Zealanders are eligible for some public funding for primary health care. To ensure that the new funding set aside for the PHCS was more likely to go initially to those most in need, the government chose to create two forms of funding – known as Access and Interim funding. Access PHOs generally served higher needs population, and were defined as those PHOs where more than 50% of the enrolled population was Maori, Pacific, or from lower socio-economic areas. All other PHOs were Interim PHOs.

PHOs are funded for first contact services via a funding formula which estimates the average number of expected primary health care visits per annum for different age groups, and, since 2002, has paid a base capitation amount of $25 per expected visit per enrolee (there are also adjustments each year to maintain the value of the subsidies). At first, Access PHOs were funded at higher capitation rates than Interim PHOs. Since 2003, the government has provided further funding, gradually increasing the capitation payment rates to Interim PHOs for particular groups in the population to the rates paid for those in Access PHOs. New funding was provided to Interim PHOs, for those aged 6–17 years of age (from 1 October 2003), those aged 65 and over (from 1 July 2004), those aged 18–24 from 1 July 2005, those aged 45–64 from 1 July 2006, and those aged 25–44 from 1 July 2007.

It was agreed that GP practices would be able to continue to set their own fees, even as the government rolled out the new funding for primary health care services. New Zealand competition law requires practices to not collude in setting patient fees. This means that practitioners have, so far, retained the right to set their own fees and thereby shape their incomes, and it also maintains a degree of competition between GPs in terms of the remaining fees faced by patients. As a result, fees continue to vary between practices. However, the government also stated that it expected that its increased capitation payments would be reflected in low or reduced costs to patients [13]. In practice, this policy was implemented through discussions between Ministry of Health officials, District Health Board (DHB) staff and PHO staff, and local PHO informal negotiations with GPs.

In Access PHOs, these discussions focused on setting 'usual' fees within specific communities and were informed by the Ministry's view that a 'low' fee should generally be a zero fee for those aged six years and under; $7–$10 for those aged 6–17; and $15–$20 for adults. In Interim PHOs and practices, for the roll out of new funding for those aged 6–17 years of age, there was a signalled desire for fees to be reduced in line with the increase in subsidies (ie a $5 increase for those with subsidy cards; $10 for those without subsidy cards). In the July 2004 roll out of new funding for those aged 65 years and over, it was expected that PHOs would reduce their charges for those people without subsidy cards by $25 and for those with subsidy cards by $10. In addition, all those eligible for the new, higher subsidy levels also became eligible for cheaper pharmaceutical services – with the patient contribution for fully subsidised items falling to $3 per prescription item.

In October 2006, a further change was made to the funding levels for PHOs, such that all those PHOs offering very low cost access (ie very low fees) became eligible for even higher levels of subsidies. At October 2006, this required zero fees for children under 6 years; a $10 maximum for children 6–17 years and a $15 maximum for all adults 18 years and over. Additional funding was provided to practices agreeing to provide 'very low cost access' from October 2007, with the aim of keeping child visits free, visits for those aged 6–17 at no more than $10.50 and adult fees at a maximum of $15.50 [14]. In January 2008, capitation payments for visits for children were increased by $6 to $45.70 where PHOs and practices agreed not to charge patients for child visits [14].

A number of other funding sources are also available for primary health care. In response to concerns that some New Zealanders with high needs, but not in Access PHOs, might continue to miss out on higher subsidies while the new funding was rolled out, and because GPs did not wish to manage the full financial risk of chronic illness from their publicly funded capitation payment and private user fees, a separate funding arrangement was established for those with chronic illnesses. Called Care Plus, this funding is targeted towards individuals who need to visit their GP or practice nurse often, due to significant chronic conditions or a terminal illness. Additional funding has been provided to support rural practice. The government has also introduced a performance management programme and funding to support clinical governance and continuous quality improvement in primary health care. Some PHOs have had further funding to support programmes to reduce inequalities, to promote innovations in nursing services, and to promote innovations in primary mental health care services [15].

Overall, the government has committed an additional $2.2 billion over the seven years from 2002/03 for implementation of the Strategy. This is a significant injection of funding for primary health care, providing around $300 million additional new funding per annum on top of an annual spend on general practitioner services of about $337 million in 2002/03 [16].

## Results

### Changes in Fees Paid by New Zealanders as a result of the Strategy

In this section we report on changes in fees brought about by the PHCS. Our focus is particularly on 'first contact services' or general medical services, the funding of which accounts for around 70% of annual capitation funding provided for primary health care [17].

**Figure 1:**
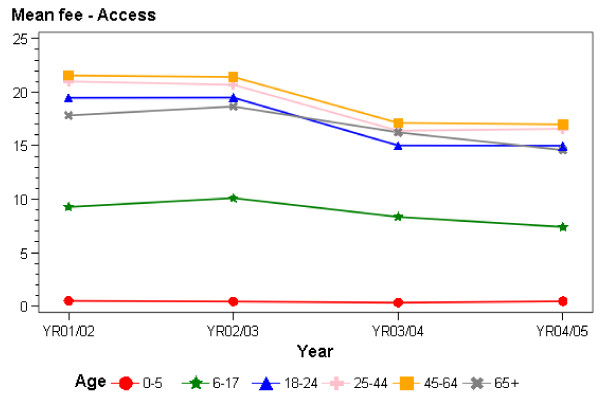
Mean patient co-payments at Access practices 2001/02-2004/05.

**Figure 2:**
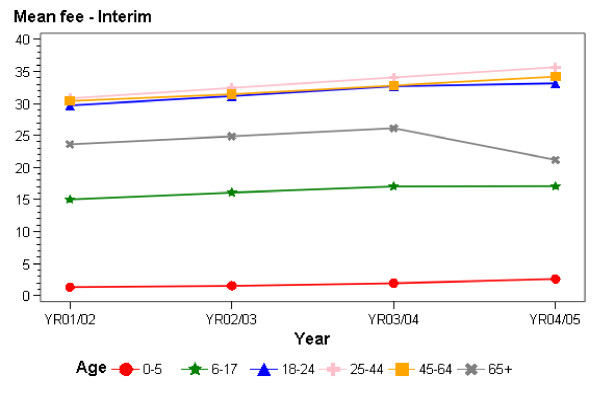
Mean patient copayments at interim practices 2001/02-2004/05.

Figures 1 and 2, and Tables 1 and 2, show the mean invoiced fees for the patients that were continually registered with a practice and the changes in mean invoiced fees over time. These fees are for general medical consultations (ie, excluding maternity, immunisations and consultations for accidents and injury, which are funded by an alternative system). All invoiced encounters, i.e. GP and nurse encounters aggregated, are included (that is, we are reporting on services provided by nurses only, by GPs only or where the patient saw both a GP and a nurse). Although explicit government policy, as discussed above, relates to the scheduled fees for doctor-only visits, the data report on actual fees paid by patients, and hence provide a picture of the way in which New Zealand patients have experienced the PHCS and the impact of new funding on the fees they pay each time they use a primary health care service. The results focus on the period between July 2001 (before the introduction of the PHCS) and mid-2005.

**Table 1:** Change in mean patient co-payments by funding model and age 2001/02-2004/05

Type	Age	2001/02	2002/03	2003/04	2004/05	Change 2001/02-2002/03	Change 2002/03-2003/04	Change 2003/04-2004/05	Change Whole Period 2001/02-2004/05
Access	0–5	0.50	0.44	0.33	0.46	-0.06 (-12%)	-0.11 (-25%)	0.13 (39%)	-0.04 (-8%)

	6–17	9.27	10.10	8.34	7.41	0.83 (9%)	-1.76 (-17%)	-0.93 (-11%)	-1.86 (-20%)

	18–24	19.47	19.49	15.02	15.00	0.02 (0%)	-4.47 (-23%)	-0.02 (0%)	-4.47 (-23%)

	25–44	21.01	20.69	16.40	16.57	-0.32 (-2%)	-4.29 (-21%)	0.17 (1%)	-4.44 (-21%)

	45–64	21.56	21.43	17.13	16.99	-0.13 (-1%)	-4.30 (-20%)	-0.14 (-1%)	-4.57 (-21%)

	65+	17.82	18.66	16.25	14.59	0.84 (5%)	-2.41 (-13%)	-1.66 (-10%)	-3.23 (-18%)

Interim	0–5	1.32	1.51	1.92	2.57	0.19 (15%)	0.41 (27%)	0.65 (34%)	1.25 (95%)

	6–17	15.01	16.07	17.02	17.07	1.06 (7%)	0.95 (6%)	0.05 (0%)	2.06 (14%)

	18–24	29.69	31.13	32.68	33.13	1.44 (5%)	1.55 (5%)	0.45 (1%)	3.44 (12%)

	25–44	30.77	32.43	34.04	35.66	1.66 (5%)	1.61 (5%)	1.62 (5%)	4.89 (16%)

	45–64	30.36	31.42	32.80	34.17	1.06 (3%)	1.38 (4%)	1.37 (4%)	3.81 (13%)

	65+	23.61	24.85	26.12	21.18	1.24 (5%)	1.27 (5%)	-4.94 (-19%)	-2.43 (-10%)

**Table 2:** Mean patient co-payments by funding model and CSC status 2001/02-2004/05

Funding Type	Age	CSC	2001/02	2002/03	2003/04	2004/05	Change 2001/02-2002/03	Change 2002/03-2003/04	Change 2003/04-2004/05	Change Whole Period 2001/02-2004/05
Access	0–5	N	0.74	0.66	0.49	0.7	-0.08 (-11%)	-0.17 (-26%)	0.21 (43%)	-0.04 (-5%)

		Y	0.37	0.32	0.25	0.33	-0.05 (-14%)	-0.07 (-22%)	0.08 (32%)	-0.04 (-11%)

	6–17	N	12.47	13.81	10.9	9.23	1.34 (11%)	-2.91 (-21%)	-1.67 (-15%)	-3.24 (-26%)

		Y	7.22	7.72	6.77	6.34	0.50 (7%)	-0.95 (-12%)	-0.43 (-6%)	-0.88 (-12%)

	18–24	N	23.2	24.6	17.44	17.09	1.40 (6%)	-7.16 (-29%)	-0.35 (-2%)	-6.11 (-26%)

		Y	17	16.57	13.6	13.85	-0.43 (-3%)	-2.97 (-18%)	0.25 (2%)	-3.15 (-19%)

	25–44	N	26.17	26.11	19.88	19.75	-0.06 (0%)	-6.23 (-24%)	-0.13 (-1%)	-6.42 (-25%)

		Y	15.93	15.57	13.24	13.91	-0.36 (-2%)	-2.33 (-15%)	0.67 (5%)	-2.02 (-13%)

	45–64	N	25.54	25.33	19.43	19.22	-0.21 (-1%)	-5.90 (-23%)	-0.21 (-1%)	-6.32 (-25%)

		Y	16.43	16.25	14.04	14.14	-0.18 (-1%)	-2.21 (-14%)	0.10 (1%)	-2.29 (-14%)

	65+	N	24.77	25	19.88	16.1	0.23 (1%)	-5.12 (-20%)	-3.78 (-19%)	-8.67 (-35%)

		Y	15.8	16.65	15.03	14.08	0.85 (5%)	-1.62 (-10%)	-0.95 (-6%)	-1.72 (-11%)

Interim	0–5	N	1.64	2	2.61	3.33	0.36 (22%)	0.61 (31%)	0.72 (28%)	1.69 (103%)

		Y	0.93	0.94	1.08	1.59	0.01 (1%)	0.14 (15%)	0.51 (47%)	0.66 (71%)

	6–17	N	16.95	18.26	19.06	18.75	1.31 (8%)	0.80 (4%)	-0.31 (-2%)	1.80 (11%)

		Y	12.5	13.26	14.43	14.91	0.76 (6%)	1.17 (9%)	0.48 (3%)	2.41 (19%)

	18–24	N	32.69	35.01	36.76	37.02	2.32 (7%)	1.75 (5%)	0.26 (1%)	4.33 (13%)

		Y	26.04	27.19	29.02	30.01	1.15 (4%)	1.83 (7%)	0.99 (3%)	3.97 (15%)

	25–44	N	33.95	35.99	37.51	39.08	2.04 (6%)	1.52 (4%)	1.57 (4%)	5.13 (15%)

		Y	24.42	25.52	27.5	29.44	1.10 (5%)	1.98 (8%)	1.94 (7%)	5.02 (21%)

	45–64	N	33.17	34.3	35.57	37.04	1.13 (3%)	1.27 (4%)	1.47 (4%)	3.87 (12%)

		Y	23.39	24.09	25.65	26.82	0.70 (3%)	1.56 (6%)	1.17 (5%)	3.43 (15%)

	65+	N	30.99	32.21	33.01	22.84	1.22 (4%)	0.80 (2%)	-10.17 (-31%)	-8.15 (-26%)

		Y	20.59	21.77	23.09	20.4	1.18 (6%)	1.32 (6%)	-2.69 (-12%)	-0.19 (-1%)

In Access practices, across the entire study period, the fall in fees for those aged under 6 years of age was around 8%, while fees have fallen for those in all the other age groups by around 20%. For those aged under six years of age, fees averaged 50 c in 2001/02, and averaged 46 c in 2004/05; for those aged 6–17, fees averaged $9.27 in 2001/2, falling to $7.41 in 2004/05. For the other age groups, fees averaged between $17.82 and $21.56 in 2001/02 and fell to between $14.59 and $16.99 in 2004/05. The range of fees by age group also narrowed.

In Interim practices, new funding had only been provided for two age groups during the time of this study (shaded in Tables 1 and 2). For those aged 18–64, fees rose slightly in each year of the study. Fees rose slightly for the first two years of the study for those aged 65 years and over, and then fell in the last year of the study as new funding was rolled out in July 2004. Fees fell from an average of $26.12 in 2003/04 to $21.18 in 2004/05, a fall of $4.94 (19%). At the end of the study period, fees in Interim practices ranged from $2.57 on average for those aged six years and under, to $17.07 for those aged 6–17, and to $21.18 for those aged 65 years and over. Fees averaged around $33–$35 for those aged 18–64 in Interim practices, that is, for the group which had yet to benefit from additional government funding at the time of data collection.

When we consider changes in the average level of fees charged by practices over time, by funding model and community services card status (Table 2), we find that different groups in the population benefited in different ways from the changes in fees brought about by the PHCS. This is unsurprising, given that some groups (those with subsidy cards) were already eligible for government funding to support primary health care prior to the introduction of the PHCS, while other groups were not.

In terms of changes in the average level of fees over time, in Access practices, there were falls of between 4 c for children with and without community services cards to falls of $8.67 for those aged 65 years and over without community services cards. Percentage falls in fees ranged from 5% for children without community services cards, to between 11% and 26% for most other population groups, to 35% for those aged 65 years and over without community services cards.

New funding was introduced for Interim practices in October 2003 for those aged 6–17 – with a $5 increase in subsidy rates for those with subsidy cards and a $10 increase in subsidy rates for those without cards. Average fees for those with cards rose slightly, while a slight fall in the average fees paid by those in this age group without cards was noticeable between 2003/04 and 2004/05 (where fees fell from an average $19.06 to $18.75; a fall of 31 c or 2%). The fall in fees was more noticeable in Interim practices following the new subsidies introduced in July 2004 for those aged 65 years and over, with fees falling by an average of $2.69 (12%) for those with cards and $10.17 on average for those without cards (a fall of 31%) between 2003/04 and 2004/05. Subsidy increases for this group (including adjustments for inflation) were $10 for those with cards and $25 (plus a $1 adjustment for inflation) for those without cards.

### Changes in consultation rates

**Figure 3:**
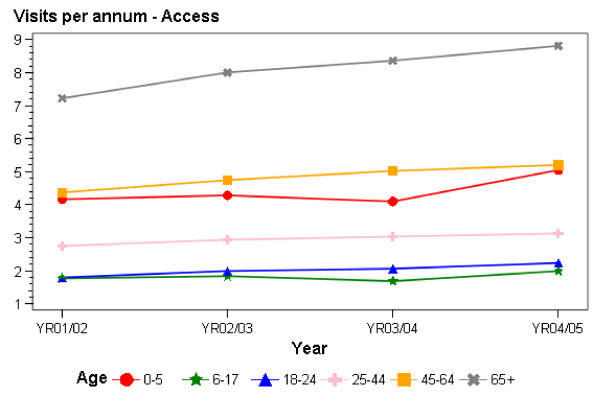
Consultation rates at Access practices 2001/02-2004/05.

**Figure 4:**
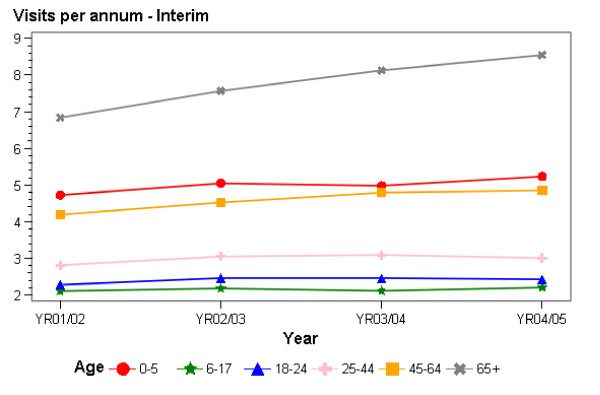
Consultation rates at Interim practices 2001/02-2004/05.

Figures 3 and 4 show the changes over time in consultation rates. The data show increases in consultation rates in Access practices across the entire study period (Figure 3). In these practices, greater increases in consultation rates occurred amongst those aged 65 years and over (1.6 consultations, 22%); 18–24 (0.4 consultations, 22%); under six (0.8 consultations, 19%) and 45–64 years of age (0.8 consultations, 18%) than in the remaining age group (25–44 years) (0.4 consultations, 15%).

In Interim practices (Figure 4), there was also an overall increase in consultation rates across the entire study period, although the increase in percentage terms was lower in Interim practices than for those in Access practices for each age group, other than for those aged 65 years and over, while being very similar in Interim and Access practices for those aged 45–64 years old. The greatest increases in consultation rates was amongst those aged 65 years and over (1.7 consultations, 25%), 45–64 (0.7, 17%) and those aged under six (0.5, 11%).

Considering the experiences of those with and without subsidy cards, there were increases over the entire study period in consultation rates for all groups, except for those aged 18–24 in Interim practices and without cards. In Access practices, percentage increases in consultation rates were highest for those aged 0–5 without cards (41%), followed by those aged 65 and over (31%), 18–24 (28%) and 6–17 (25%) without cards. Within each age group, increases in consultation rates were higher for those without cards. In Interim practices, increases in consultation rates were highest for those in the older age groups (those aged 65 years and over without (28%) and with cards (26%) and those aged 45–64 without cards (22%)). Increases in consultation rates were slightly higher for those without cards than for those with cards for those aged 0–5 years of age, 6–17 years of age, and 65 years and over, while those with cards had higher rates of increase than those without cards in the 18–64 years age group.

## Discussion

### Main findings in relation to government objectives: fees

As a result of the additional public funding for primary health care, fees fell particularly in Access practices for doctor and nurse visits. Fees have increased over time for many in Interim practices, but they fell for those aged 65 years and over as new funding was provided for this age group in Interim practices. Thus, at one level, the government's goal of increasing funding to PHOs in order to reduce the fees patients pay was achieved – fees generally fell where one would have expected them to fall (for those in Access practices and for those aged 65 years and over in Interim practices).

For patients in Access practices, and for those in Interim practices aged 6–17 and 65 years and over (who were eligible for the extra public funding for longest), a key question is whether the changes in fees met the scale of fee reductions the government was looking for. The general expectation was that fees should be 'low' or reduce as new funding became available. In Access practices, the government was looking for a zero fee for those aged six years and under; $7–$10 for those aged 6–17; and $15–$20 for adults. Fees clearly fell in Access practices over the study period. By the end, average fees in Access practices were 46 c for those six years and under; $7.41 for those aged 6–17; and below $20 for adults. Thus, the government's policy objectives were close to being met for younger children in Access practices and were being met for those aged 6–17 years and for adults.

In terms of Interim practice patients, the data show that the new funding to those aged 6–17 led to only a small reduction in the overall fees paid by those without subsidy cards and a small increase in fees paid by those with cards. Part of the explanation for fees not reducing much in this group relates to the fact that the new funding came to only around $5 (for those with cards) to $10 (for those without cards) per consultation, as before the PHCS those in this age group had already received a government subsidy for primary health care of $20 and $15, respectively. Reductions in fees for those aged 65 years and over were much greater: in the year after they became eligible, fees for doctor and nurse visits fell by an average of $10.17 (31%) for those aged 65 years and over without cards and $2.69 (12%) for those with cards. The government had been seeking greater falls in schedule fees of around $25 for those without cards and of $10 for those with cards.

In interpreting these findings, it is important to remember that the data relate to the fees charged to patients, whereas government policy focused on schedule fees; i.e. the fees that are set out in schedules and displayed in offices to inform patients of the fees charged for standard consultations. If scheduled fees did fall by the amounts required by the government, our findings reflect considerable differences between scheduled and actual fees charged to patients. This may arise from consultations being shorter than the standard consultation assumed to calculate the scheduled fees, or from greater nurse or other provider involvement in consultations than for a standard consultation, or as a result of significant amounts of pre-PHCS discounting of fees by general practices. Our findings also relate to both doctor and nurse visits, while the explicit government policy was focused on doctor visits only; however, additional analyses using data on doctor visits only shows a similar pattern of changes in fees as for doctor and nurse visits combined (not shown) [18].

Although the government signalled that it wished to see fees for those with and without subsidy cards to be the same, our results also show that those with cards were charged lower fees than those without cards in both Access and Interim practices (Table 2). This is likely to represent the persistence of longstanding practitioner behaviour under the previous targeted subsidy regime in which practitioners took account of what they supposed to be the financial circumstances of different patients when charging them. The continuation of such behaviour may work in an unplanned way towards reducing inequalities in access to health services (and thence perhaps to reduced health inequalities) by enabling even cheaper access to care for more disadvantaged groups. On the other hand, the reductions in fees, as opposed to the total fees charged, have tended to benefit those without the previous subsidy cards. In Access practices, much of the benefit of the new funding is, as expected, going to those without subsidy cards while in Interim practices, fees are generally not rising as fast for those without cards, i.e. benefiting those in better socio-economic positions (except for children). For example, fees have fallen further for those aged 65 years and over without cards. This is because the aim of policy since 2001 has been to relate public funding for primary health care more closely to those who need care rather than to target it on those with the lowest incomes or highest level of past use as under the pre-PHCS approach.

With almost all practices becoming members of PHOs and with almost all New Zealanders now enrolled in a PHO, there is no definitive way of knowing how fees might have changed in the absence of increased funding for primary health care. Recent research by Cumming and Stillman (personal communication, J Cumming and S Stillman) shows that nominal fees paid by patients rose by 40% between 1996/97 and 2002/03, i.e. at around 6% per annum, across all population groups. Assuming that the 1996/97-2002/03 period is typical of trends in the costs of general practice services before the PHCS was implemented, we might have expected fees to have risen by around 6% per annum in the absence of the PHCS, or by around 18% over the 2001/02-2004/05 period. Instead, the results show that fees fell in Access practices as they became part of PHOs by between 8% and 20% between 2001/02 and 2004/05, when we might have expected fees to have risen over this period by around 18%.

In Interim practices, other than for children, fee increases across the study period were broadly within the likely 6% per annum increase that would have been expected without the PHCS, though fee increases were possibly lower than might have been expected between 2002/03 and 2003/04. However, over the entire period, fees fell to the extent that might have been expected for those aged 6–17, while they fell for those aged 65 years and over (by between 12% and 33%, when an overall increase of about 18% might have been expected). All the fee reductions or reduced rates of increase were achieved with the injection of a large amount of new public funding.

Of particular interest in these analyses is the overall impact of the changes on provider incomes, particularly GP practice incomes. Government policy has tended to tighten over time in order to alleviate concerns that the new funding could have resulted not in lower fees for patients, but in higher incomes for providers. The evidence reported here does show that not all the increase in government funding was reflected in reduced fees for patients. Survey evidence from other parts of our evaluation of the PHCS shows that 72% of practices sampled (n = 276) reported an increase in their income since joining a PHO (compared with 7% which reported a decrease), with higher incomes more likely to be reported by Interim practices (83%) (whose enrolees were not yet all entitled to government subsidies for primary health care) compared with Access practices (51%) (whose populations were by then fully covered by the new funding arrangements) [19]. Other evidence from a smaller sample of general practices similarly shows an increase in median nominal net profit per working owner from $97,220 in 2002 (n = 114) to $153,886 in 2005 (n = 79) [20,21]. Some commentators have argued, however, that this increase in incomes is no bad thing, given previous concerns over the recruitment and retention of GPs in New Zealand.

It may also be the case that the increase in public funding is not exactly reflected in reduced patient out-of-pocket fees for other reasons. The length or nature of consultations may have changed over time; new funding could be being used to pay for higher overall practice costs; or the number of visits and workloads of practices may have increased. Indeed, 73% of practices in the above practice survey also reported being busier than they had been before joining a PHO [19] and the research reported in the current article also shows an increase in consultation rates (in part related to the decrease in user fees), so that some of the increase in incomes may well be as a result of increased work undertaken by practices.

### Main findings in relation to government objectives: consultation rates

In terms of consultations, it appears that the government's aim of increasing consultation rates for primary health care was achieved. There were increases in consultation rates across almost all age, funding model, subsidy card and ethnic groups. Increases were particularly high in Access practices, especially for those without community services cards; and for those aged 65 years with and without community services cards and those aged 45–64 with community services cards in Interim practices. Consultation rates increased for all ethnic groups, with similar increases for Pacific, Maori, and European and 'Other' ethnic groups, and smaller increases for Asian populations.

The overall increase in average consultation rates appears to be reasonable in terms of the number of consultations, with increases of over 20% for some in Access practices. Increases in Interim practices were generally lower, as might have been expected given that new funding had not been allocated to all groups in Interim practices throughout the study period, although consultation rates in Interim practices increased by more than 20% for those aged 45–64 without community cards and for those aged 65 years and over with and without cards.

The increase in consultation rates does seem to be associated with a reduction in unmet need for primary health care. Recent evidence from the 2006/07 New Zealand Health Survey shows that New Zealanders' unmet need, as demonstrated by the percentage of people reporting wanting to visit a primary health care provider but not being able to do so, has fallen over the period since the implementation of the Strategy began, from 12% in 2002/03 to 6.3% in 2006/07 [22,23].

## Conclusion

New Zealand's Primary Health Care Strategy resulted in lower fees for primary health care for many New Zealanders, and consultation rates also increased. However, fees did not fall by as much as expected in government policy statements, and greater reductions in fees occurred in groups in the population who might be considered to be in a better financial position.

The government now needs to find a way to assess the benefits of the improved access and higher utilisation it has obtained from the substantial increase in its expenditure over the past few years to assure itself that the Strategy is providing value for money, and contributing to improving health and reducing health inequalities as originally intended. Then the government needs to develop the payment system to encourage the provision of more primary care that improves health and reduces inequalities.

Another key issue for the government is how to explicitly define and maintain low fees (i.e. fee levels that do not deter any sub-group in the population from making timely, effective use of first contact primary health care) when it does not fully fund primary health care, and while ensuring that public funding legitimately compensates for the increasing costs faced by health professionals and provides them with a reasonable, but not excessive take-home income sufficient to ensure an adequate supply of medical and nursing staff across the country. Compared with earlier policy settings, New Zealand now has better mechanisms to manage the fees that patients pay, including fees review processes where local fees are perceived to be too high and the agreed capping of fees where providers accept higher levels of capitation payment. However, there are limited requirements for practitioners to pass on increased public funding to patients in the shape of reduced user fees. By contrast, providers will have concerns about the impact of fee agreement and review processes on their ability to set fees and hence to recover increasing costs, earn higher incomes (including where they do extra work) and maintain the value of their businesses. As a result, each new allocation of public funding is likely to result in policy debate over the balance between maintaining or reducing patient fees and rewarding professionals for their contribution to New Zealanders' health.

## Methods

This article is focused on the period from June 2001 until mid – 2005. It provides data on fees and use of services covered by:

• the year before the first PHO was established in July 2002

• the roll out of new funding for Access PHOs as they were established after July 2002

• the roll out of new funding to Interim PHOs as they were established, and

• the roll out of new funding to Interim PHOs for those aged 6–17 in October 2003 and those aged 65 years and over in July 2004.

While government policy during the period focused on changes in scheduled fees, that is on the fees doctors advertised for standard consultations, the analyses here focus on fees actually paid by patients for their consultations with both doctors and nurses (for doctor consultations, we see a similar pattern of fees, not reported here) [18].

The research uses a before-and-after design to explore the changes occurring in fees and consultation rates. A power analysis showed that a sample of 100 general practices would provide adequate power to address key research questions, based upon known distributions of variables of interest. A national sample of 100 practices was drawn from lists of all currently active practices that are members of PHOs. Practices were invited to take part in the research, and paid $250 for participation. Practices were offered the opportunity to receive analyses comparing their fees and, patterns of utilisation with all other practices sampled. Overall, 100 out of 115 practices approached to participate did so (87%). However, data from one practice proved to be unuseable, leaving a sample of 99 practices.

The total population of registered patients in the final sample was 421,993, or 10.4% of the NZ population. As a result of some practices not participating in the research, the sample over-sampled Access practices.

The data collected were:

► A register download of registration status, date of birth, gender, ethnicity, deprivation code, current subsidy card status.

► Dates of consultations since 1 June 2001.

► The practitioner (doctor or nurse) seen at each encounter.

► Information on the fees charged to patients for each consultation.

A number of technical issues also arose in undertaking these analyses. First, when reporting co-payments, there is a wide range of services provided by primary health care providers. Some minor surgical procedures cost hundreds of dollars (e.g. a vasectomy); other invoices show negative amounts, corresponding to a refund being issued to a patient. To eliminate the impact of extreme outliers, the co-payments data were censored, restricting co-payments to values between $0 and $100. Second, we needed to recognise the difference between invoiced encounters and encounters for which there was no associated invoice. All data presented in the graphs and tables are for invoiced encounters only (which includes invoices where the charge is $0). For an invoiced encounter to be recorded when a patient fee is not charged, a "zero invoice" must be entered. This is typically for visits by young children or for people who use a lot of services, such as the elderly or patients with chronic conditions, and for visits to a practice nurse. However, "zero invoice" information is not entered reliably into practice management systems, and data collections based on invoiced encounters may therefore tend to underestimate overall consultation rates.

Ethics Approval was given for the study by the New Zealand Multi Region Ethics Committee. A Memorandum of Understanding was signed between the researchers and each participating practice, describing data collection and analysis procedures.

## Limitations of the study

First, we have no way of taking account of the content of consultations. We censored the fees data in order to reduce the impact of higher cost services on the analyses, but even then, the fees reported may represent a wide range of different types of consultations, including the provision of special procedures or a number of services at one visit, or a longer consultation. The estimates of changes over time in relation to fees and consultations also assume that there was no change in the nature or length of consultations.

Second, not all encounters have an associated invoice and we may have under-estimated the number of consultations taking place as a result of this (e.g., consultations for young children, nurses visits, or visits by high users, as well as consultations for repeat prescriptions attract an invoice). We may therefore have over-estimated the average fees paid by patients, as encounters where there is no invoice generated have not been included in the calculations of average fees (although encounters where a zero invoice was noted are included).

Third, of necessity, there is no control group with which to compare experiences. We cannot be sure what might have happened to both fees and consultation rates in the absence of the PHCS and of new funding allocated by government to primary health care. We have indicated how fees may have changed in the absence of the PHCS, but this can only be an estimate.

Fourth, our findings are based on an analysis of data from 99 general practices. The sample slightly over-represents Access practices. We have also had to limit the data to patients who were registered with a particular practice at the time of this analysis. Patients who shifted between practices may differ. Our analyses by subsidy card population groups are also dependent on data availability. The analyses here assign a subsidy card to anyone who ever held a card. The analyses may therefore classify some people who no longer have or are eligible for cards within the card-holding group.

Finally, there are potential gaps in our dataset. There may be some services which are not recorded here, either because they are organised by the PHOs or because not all services delivered by practices are recorded. Changes in recording may also impact on our findings (for example, nursing services may simply be being recorded more).

## Competing interests

NM was a principal advisor in the social policy branch of the New Zealand Treasury, 1998–2003. Between 2004 and 2007, he worked for the Treasury for three months of the year. In 2008, he worked for the Ministry of Health for three months. At the Treasury, he advised on primary care policy, but the views expressed here do not reflect the views of the Treasury or the Ministry of Health.

## Authors' contributions

JC was involved in the design and management of the study, analysis and interpretation of findings, and preparation of this manuscript. NM helped to interpret the findings and draft the manuscript. BG was responsible for data collection and statistical analyses and provided comments on drafts of the article. All authors read and approved the final manuscript.
